# A Stochastic Restricted Principal Components Regression Estimator in the Linear Model

**DOI:** 10.1155/2014/231506

**Published:** 2014-01-23

**Authors:** Daojiang He, Yan Wu

**Affiliations:** Department of Statistics, Anhui Normal University, Wuhu 241000, China

## Abstract

We propose a new estimator to combat the multicollinearity in the linear model when there are stochastic linear restrictions on the regression coefficients. The new estimator is constructed by combining the ordinary mixed estimator (OME) and the principal components regression (PCR) estimator, which is called the stochastic restricted principal components (SRPC) regression estimator. Necessary and sufficient conditions for the superiority of the SRPC estimator over the OME and the PCR estimator are derived in the sense of the mean squared error matrix criterion. Finally, we give a numerical example and a Monte Carlo study to illustrate the performance of the proposed estimator.

## 1. Introduction

In linear regression analysis, the presence of multicollinearity among regressor variables may cause highly unstable least squares estimates of the regression parameters. With multicollinear data, some coefficients may be statistically insignificant and may have the wrong signs. To overcome this problem, different remedial methods have been proposed. One estimation technique designed to combat collinearity is using biased estimators, most notable of which are the Stein estimator by Stein [1], the principal components regression (PCR) estimator by Massy [2], the ordinary ridge regression (ORR) estimator by Hoerl and Kennard [3], and the Liu estimator by Liu [4]. Another method to combat multicollinearity is through the collection and use of additional information, which can be exact or stochastic restrictions [5]. When it comes to stochastic linear restrictions, Durbin [6], Theil and Goldberger [7], and Theil [8] proposed the ordinary mixed estimator (OME) by combining the sample model with stochastic restrictions. Some other important references on this subject are Li and Yang [9, 10], Xu and Yang [11], Yang and Cui [12], Yang and Wu [13], Yang and Xu [14], and so on.

In this paper, we will introduce a stochastic restricted principal components (SRPC) regression estimator, which is defined by combining in a special way the ordinary mixed estimator and the principal components regression estimator. We will compare the new estimator with the PCR estimator and the OME, respectively, in the sense of the criterion of the mean squared error matrix (MSEM).

The rest of the paper is organized as follows. In Section 2, the new estimator is introduced. In Section 3, some properties of the new estimator are discussed. A numerical example and a Monte Carlo simulation study are given in Section 4.

## 2. The New Estimator

Let us consider the general linear model

(1) $$\begin{array}{c} Y=X\beta +ɛ,\;\;E\left(ɛ\right)=0,\;\;\text{Cov}\left(ɛ\right)=\sigma^{2}I_{n}, \end{array}$$

where *Y* is an *n* × 1 observable random vector with the expectation *E*(*Y*) = *Xβ* and the covariance matrix Cov(*Y*) = *σ* ^2^ *I*_*n*_, *X* is an *n* × *p* known design matrix of rank *p*, *I*_*n*_ is the identity matrix of order *n*, *β* is a *p* × 1 vector of unknown parameters, and *ɛ* is an *n* × 1 vector of random errors. As is well known, the ordinary least squares estimator (OLSE) of *β* is

(2) $$\begin{array}{c} {\hat{\beta }}_{\text{OLSE}}=S^{-1}X^{\prime }Y, \end{array}$$

where *S* = *X*′*X*.

Let *T* = (*t*_1_, *t*_2_,…, *t*_*p*_) be an orthogonal matrix such that *T*′*X*′*XT* = Λ, where Λ = diag⁡(*λ*_1_, *λ*_2_,…, *λ*_*p*_) and *λ*_1_ ≥ *λ*_2_ ≥ ⋯≥*λ*_*p*_ > 0 are the eigenvalues of *X*′*X*. Further, let *T*_*k*_ = (*t*_1_, *t*_2_,…, *t*_*k*_) be the remaining columns of *T* after having deleted the last *p* − *k* columns, where 0 ≤ *k* ≤ *p*. Thus, we have

(3) $$\begin{array}{c} T_{k}^{\prime }X^{\prime }XT_{k}=\Lambda_{k}=\mathrm{diag}\left(\lambda_{1},\lambda_{2},\ldots ,\lambda_{k}\right),\\ T_{p-k}^{\prime }X^{\prime }XT_{p-k}=\Lambda_{p-k}=\mathrm{diag}\left(\lambda_{k+1},\lambda_{k+2},\ldots ,\lambda_{p}\right), \end{array}$$

where *T*_*p*−*k*_ = (*t*_*k*+1_, *t*_*k*+2_,…, *t*_*p*_).

In addition to model (1), let us give some prior information about *β* in the form of a set of *j* independent stochastic linear restrictions as follows:

(4) $$\begin{array}{c} r=R\beta +\upsilon ,\;\;E\left(\upsilon \right)=0,\;\;\text{Cov}\left(\upsilon \right)=\sigma^{2}W, \end{array}$$

where *r* is a *j* × 1 vector, *R* is a *j* × *p* matrix with rank (*R*) = *j*, *υ* is a *j* × 1 vector of disturbances, and *W* is assumed to be known and positive definite. Furthermore, it is also assumed that the random vector *υ* is independent of *ɛ*.

For model (1), Massy [2] introduced the PCR estimator as

(5) $$\begin{array}{c} {\hat{\beta }}_{\text{PCR}}=T_{k}{\left(T_{k}^{\prime }ST_{k}\right)}^{-1}T_{k}^{\prime }X^{\prime }Y. \end{array}$$

Xu and Yang [11] showed that the PCR estimator could be rewritten as follows:

(6) $$\begin{array}{c} {\hat{\beta }}_{\text{PCR}}=T_{k}T_{k}^{\prime }{\hat{\beta }}_{\text{OLSE}}. \end{array}$$

For model (1) with the stochastic restrictions (4), the OME is given by

(7) $$\begin{array}{c} {\hat{\beta }}_{\text{OME}}={\left(S+R^{\prime }W^{-1}R\right)}^{-1}\left(X^{\prime }Y+R^{\prime }W^{-1}r\right). \end{array}$$

Özkale [15] showed that the OME could be rewritten as

(8) $$\begin{array}{c} {\hat{\beta }}_{\text{OME}}={\hat{\beta }}_{\text{OLSE}}+S^{-1}R^{\prime }{\left(W+RS^{-1}R^{\prime }\right)}^{-1}\left(r-R{\hat{\beta }}_{\text{OLSE}}\right). \end{array}$$

Noting that *T*_*k*_′*T* = (*I*_*k*_⋮0)*T*′*T* = (*I*_*k*_⋮0), it can be shown that

(9) $$\begin{array}{c} {\hat{\beta }}_{\text{PCR}}=T_{k}{\left(T_{k}^{\prime }ST_{k}\right)}^{-1}T_{k}^{\prime }X^{\prime }Y=S^{-1}T_{k}T_{k}^{\prime }X^{\prime }Y. \end{array}$$

Now, the stochastic restricted principal components (SRPC) regression estimator can be obtained by combing the OME and PCR estimator. Substituting OLSE with PCR estimator in (8), we can get the new estimator as follows:

(10) β^SRPC=β^PCR+S−1R′(W+RS−1R′)−1(r−Rβ^PCR)=S−1TkTk′X′Y+S−1R′(W+RS−1R′)−1 ×(r−RS−1TkTk′X′Y)=(S−1−S−1R′(W+RS−1R′)−1RS−1)     ×(TkTk′X′Y+R′W−1r)=(S+R′W−1R)−1(TkTk′X′Y+R′W−1r).

Now, we can see that ${\hat{\beta }}_{\text{SRPC}}$ is a general estimator which includes the PCR estimator and OME as special cases: if *R* = 0, then ${\hat{\beta }}_{\text{SRPC}}={\hat{\beta }}_{\text{PCR}}$; if *k* = *p*, then ${\hat{\beta }}_{\text{SRPC}}={\hat{\beta }}_{\text{OME}}$.

For the sake of convenience, we list some notations and important lemmas needed in the following discussions. For an *n* × *n* matrix *M*, *M* ≥ 0 means that *M* is symmetric and positive semidefinite and *M* > 0 means that *M* is symmetric and positive definite.

Note that for any estimator $\hat{\beta }$ of *β*, its MSEM is defined as

(11) MESM(β^)=E[(β^−β)(β^−β)′]=Cov(β^)+Bias(β^)Bias(β^)′,

where $\text{Bias}(\hat{\beta })=E(\hat{\beta })-\beta$ is the bias of $\hat{\beta }$.

**Lemma 1**

Let *M* > 0, and *α* a vector; then *M* − *αα*′ ≥ 0 if and only if *α*′*M* ^−1^ *α* < 1.

**Proof**

See Farebrother [16].

By Lemma 1, the following lemma is straightforward.

**Lemma 2**

Let ${\hat{\beta }}_{1}=A_{1}Y$, ${\hat{\beta }}_{2}=A_{2}Y$ be two homogeneous linear estimators of *β* such that *D* : = (*A*_1_ *A*_1_′ − *A*_2_ *A*_2_′) > 0. Then

(12) $$\begin{array}{c} MSEM\left({\hat{\beta }}_{1}\right)-MSEM\left({\hat{\beta }}_{2}\right)=\sigma^{2}D+b_{1}b_{1}^{\prime }-b_{2}b_{2}^{\prime }>0 \end{array}$$

if and only if

(13) $$\begin{array}{c} b_{2}^{T}{\left(\sigma^{2}D+b_{1}b_{1}^{\prime }\right)}^{-1}b_{2}<1, \end{array}$$

where $b_{i}=Bias({\hat{\beta }}_{i})=(A_{i}X-I)\beta$, *i* = 1,2.

**Lemma 3**

Let *M*, *N* be two *n* × *n* matrices with *M* > 0 and *N* ≥ 0; then *M* > *N*⇔*λ*_max⁡_(*NM* ^−1^) < 1.

**Proof**

See Rao and Toutenburg [5].

## 3. The Superiority of the New Estimator

The bias vector and the covariance matrix of the SRPC estimator are given by

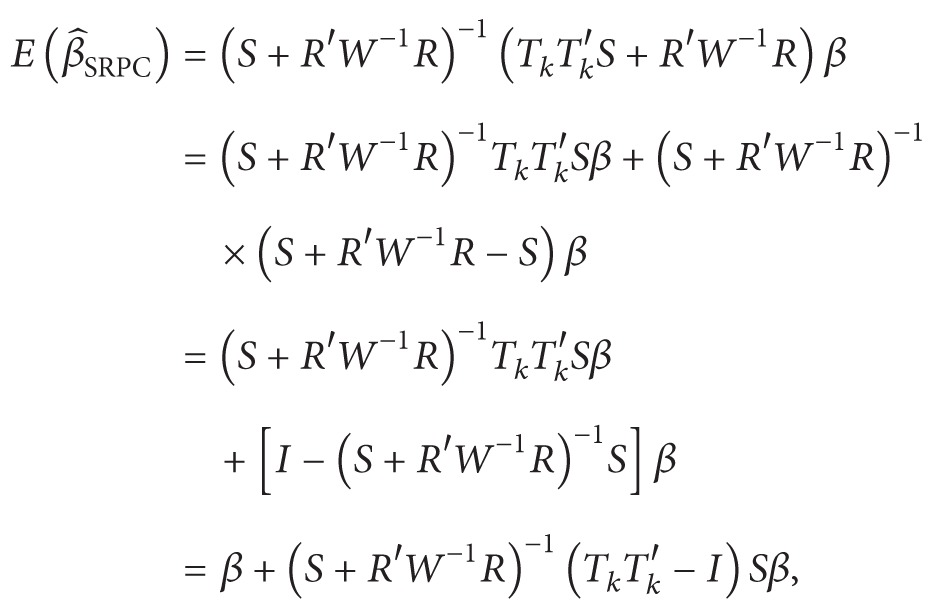
(14)

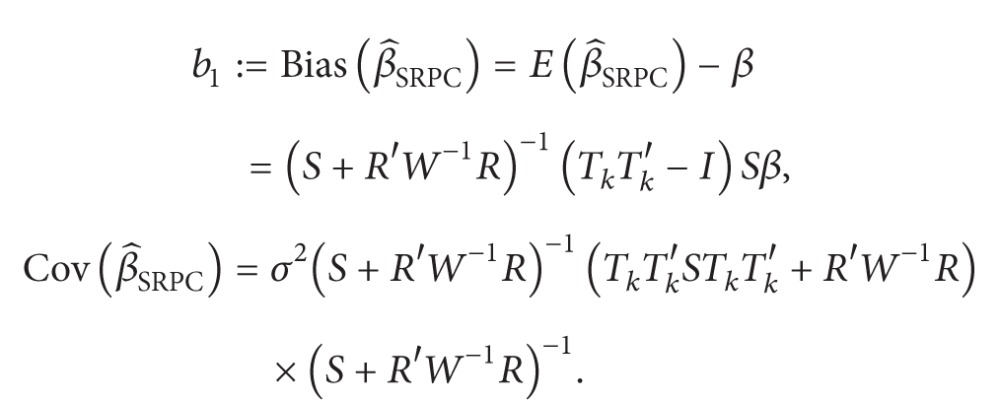
(15)
From (15), we can obtain that

(16) MSEM(β^SRPC) =σ2(S+R′W−1R)−1(TkTk′STkTk′+R′W−1R)  ×(S+R′W−1R)−1+b1b1′.

Following the above procedure, we can get

(17) $$\begin{array}{c} \text{MSEM}\left({\hat{\beta }}_{\text{PCR}}\right)=\sigma^{2}T_{k}\Lambda_{k}^{-1}T_{k}^{\prime }+b_{2}b_{2}^{\prime },\\ \text{MSEM}\left({\hat{\beta }}_{\text{OME}}\right)=\sigma^{2}{\left(S+R^{\prime }W^{-1}R\right)}^{-1}, \end{array}$$

where *b*_2_ = (*T*_*k*_ *T*_*k*_′ − *I*_*p*_)*β*.

In order to compare ${\hat{\beta }}_{\text{SRPC}}$ with ${\hat{\beta }}_{\text{PCR}}$ and ${\hat{\beta }}_{\text{OME}}$ in the MSEM sense, now we investigate the following differences:

(18) Δ1=MSEM(β^PCR)−MSEM(β^SRPC)=σ2[TkΛk−1Tk′−(S+R′W−1R)−1   ×(TkTk′STkTk′+R′W−1R)(S+R′W−1R)−1] +b2b2′−b1b1′∶=σ2D1+b2b2′−b1b1′,

where

(19) D1=TkΛk−1Tk′−(S+R′W−1R)−1 ×(TkTk′STkTk′+R′W−1R)(S+R′W−1R)−1,Δ2=MSEM(β^OME)−MSEM(β^SRPC)=σ2(S+R′W−1R)−1−σ2(S+R′W−1R)−1 ×(TkTk′STkTk′+R′W−1R) ·(S+R′W−1R)−1−b1b1′=σ2(S+R′W−1R)−1(S−TkTk′STkTk′) ×(S+R′W−1R)−1−b1b1′∶=σ2(S+R′W−1R)−1D2(S+R′W−1R)−1−b1b1′,

where

(20) $$\begin{array}{c} D_{2}=S-T_{k}T_{k}^{\prime }ST_{k}T_{k}^{\prime }. \end{array}$$

In the following theorems, we will give the necessary and sufficient conditions for the new estimator to be superior to the PCR estimator and OME in the MSEM sense.

**Definition 4**

Suppose that ${\hat{\beta }}_{1}$ and ${\hat{\beta }}_{2}$ are two estimators of *β*; then ${\hat{\beta }}_{1}$ is said to be superior to ${\hat{\beta }}_{2}$ in the MSEM sense if $\text{MSEM}({\hat{\beta }}_{2})-\text{MSEM}({\hat{\beta }}_{1})>0$.

**Theorem 5**

Assume that *λ*_max⁡_(FTΛT′) < 1; then the SRPC estimator ${\hat{\beta }}_{SRPC}$ is superior to the PCR estimator ${\hat{\beta }}_{PCR}$ in the MSEM sense if and only if

(21) $$\begin{array}{c} b_{1}^{\prime }{\left(\sigma^{2}D_{1}+b_{2}b_{2}^{\prime }\right)}^{-1}b_{1}<1, \end{array}$$

where

(22) F=(S+R′W−1R)−1(TkTk′STkTk′+R′W−1R) ×(S+R′W−1R)−1+Tp−kΛp−k−1Tp−k′.

**Proof**

Noting that *T*Λ^−1^ *T*′ = *T*_*k*_Λ_*k*_^−1^ *T*_*k*_′ + *T*_*p*−*k*_Λ_*p*−*k*_^−1^ *T*_*p*−*k*_′, it follows that

(23) D1=TkΛk−1Tk′−(S+R′W−1R)−1 ×(TkTk′STkTk′+R′W−1R)(S+R′W−1R)−1=TΛ−1T′−F.

By Lemma 3 and the assumption that *λ*_max⁡_(*FT*Λ*T*′) < 1, we have *D*_1_ > 0. Consequently, by Lemma 2, Δ_1_ = *σ* ^2^ *D*_1_ + *b*_2_ *b*_2_′ − *b*_1_ *b*_1_′ > 0 if and only if

(24) $$\begin{array}{c} b_{1}^{\prime }{\left(\sigma^{2}D_{1}+b_{2}b_{2}^{\prime }\right)}^{-1}b_{1}<1. \end{array}$$

Thus, the proof of Theorem 5 is completed.

**Theorem 6**

Assume that *λ*_max⁡_(*T*_*k*_ *T*_*k*_′*ST*_*k*_ *T*_*k*_′*S* ^−1^) < 1; then the SRPC estimator ${\hat{\beta }}_{SRPC}$ is superior to the the mixed estimator ${\hat{\beta }}_{OME}$ in the MSEM sense if and only if

(25) $$\begin{array}{c} b_{1}^{\prime }{\left({\left(S+R^{\prime }W^{-1}R\right)}^{-1}D_{2}{\left(S+R^{\prime }W^{-1}R\right)}^{-1}\right)}^{-1}b_{1}<\sigma^{2}. \end{array}$$

**Proof**

By Lemma 3, the assumption that *λ*_max⁡_(*T*_*k*_ *T*_*k*_′*ST*_*k*_ *T*_*k*_′*S* ^−1^) < 1 implies that *D*_2_ = *S* − *T*_*k*_ *T*_*k*_′*ST*_*k*_ *T*_*k*_′ > 0. It follows that

(26) $$\begin{array}{c} {\left(S+R^{\prime }W^{-1}R\right)}^{-1}D_{2}{\left(S+R^{\prime }W^{-1}R\right)}^{-1}>0. \end{array}$$

Therefore, Δ_2_ = *σ* ^2^(*S* + *R*′*W* ^−1^ *R*)^−1^ *D*_2_(*S* + *R*′*W* ^−1^ *R*)^−1^ − *b*_1_ *b*_1_′ > 0 if and only if

(27) $$\begin{array}{c} b_{1}^{\prime }{\left({\left(S+R^{\prime }W^{-1}R\right)}^{-1}D_{2}{\left(S+R^{\prime }W^{-1}R\right)}^{-1}\right)}^{-1}b_{1}<\sigma^{2}. \end{array}$$

## 4. Numerical Example and Monte Carlo Simulation

In order to illustrate the performance of the proposed estimator, we first consider the real data example which was discussed in Gruber [17], and the data has also been analyzed by Akdeniz and Erol [18], Li and Yang [9], and Chang and Yang, [19] among others. Now we assemble the data as follows:

(28) $$\begin{array}{c} X=\left(\begin{array}{ccccc} 1 & 1.9 & 2.2 & 1.9 & 3.7\\ 1 & 1.8 & 2.2 & 2.0 & 3.8\\ 1 & 1.8 & 2.4 & 2.1 & 3.6\\ 1 & 1.8 & 2.4 & 2.2 & 3.8\\ 1 & 2.0 & 2.5 & 2.3 & 3.8\\ 1 & 2.1 & 2.6 & 2.4 & 3.7\\ 1 & 2.1 & 2.6 & 2.6 & 3.8\\ 1 & 2.2 & 2.6 & 2.6 & 4.0\\ 1 & 2.3 & 2.8 & 2.8 & 3.7\\ 1 & 2.3 & 2.7 & 2.8 & 3.8 \end{array}\right),\\ Y=\left(\begin{array}{c} 2.3\\ 2.2\\ 2.2\\ 2.3\\ 2.4\\ 2.5\\ 2.6\\ 2.6\\ 2.7\\ 2.7 \end{array}\right). \end{array}$$

In this experiment, we can note from the theorems that the comparison results depend on the unknown parameters *β* and *σ* ^2^. Consequently, we cannot exclude that our obtained results in the theorems will be held and the results may be changeable. For this, we replace them by their unbiased estimators, that is, the OLS estimators. The results below are all computed by R2.8.0.

From the data, we can obtain the following results:

- the eigenvalues of *X*′*X*: 312.9320, 0.7536, 0.0453, 0.0372, and 0.0019.
- the OLS estimator of *β*: (0.6921,0.6258,0.1154,0.2866, and  0.0256)′  ,
- the OLS estimator of *σ* ^2^: ${\hat{\sigma }}^{2}=0.0016$,
- the condition number of *X*′*X*: *κ* = 1.6107*e* + 005.

Following Chang and Yang [19], we choose the number of the principal components *k* = 3, and consider the following stochastic linear restriction:

(29) $$\begin{array}{c} r=R\beta +e,\;R=\left(1,1,2,-2,-2\right),\;e\sim N\left(0,{\hat{\sigma }}^{2}\right). \end{array}$$

The estimated MSE values of PCR, OME, and SRPC are obtained by replacing all unknown parameters by their OLS estimators, respectively.  Table 1 gives the results.

From Table 1, we can observe that the estimated MSE value of the new estimator is smaller than those of PCR and OME, which is in accordance with the theoretical findings in Theorems 5 and 6.

To further identify the MSE performance of the new estimator, we are to perform a Monte Carlo simulation study. Specifically, the explanatory variables and the observations are generated by

(30) xij=(1−γ2)1/2ωij+γωi,p, i=1,2,…,n,             j=1,2,…,p−1,yi=β1xi1+β2xi2+β3xi3+β4xi4+β5xi5+ei,        ɛi~N(0,σ2), i=1,2,…,n,

where *ω*_*ij*_ are independent standard normal pseudorandom numbers, *ɛ*_*i*_ are independent normal pseudorandom numbers with mean zero and variance *σ* ^2^, and *γ* is specified so that the correlation between any two explanatory variables is given by *γ* ^2^. In addition, a stochastic linear constraint to the model is considered:

(31) $$\begin{array}{c} r=R\beta +e,\;R=\left(1,3,-2,-2,1\right),\;e\sim N\left(0,W\right). \end{array}$$

In the simulation, we choose *σ* ^2^ = 1, *p* = 6, *n* = 50, 100, and *W* = 2, 5. Four different sets of correlations, namely, *γ* = 0.9, *γ* = 0.99, *γ* = 0.999, and *γ* = 0.9999, are considered to show the weak, strong, and severe collinearity between the explanatory variables following Liu [20]. We choose the normalized eigenvector corresponding to the largest eigenvalue of *X*′*X* as the true value of *β* following Chang and Yang [19]. The experiment is replicated 10000 times by generating new error terms. Then, the estimated MSE for an estimator $\tilde{\beta }$ is calculated as follows:

(32) $$\begin{array}{c} \text{MSE}\left(\tilde{\beta }\right)=\frac{1}{N}\overset{N}{\underset{m=1}{\sum }}{\left({\tilde{\beta }}^{\left(m\right)}-\beta \right)}^{\prime }\left({\tilde{\beta }}^{\left(m\right)}-\beta \right), \end{array}$$

where ${\tilde{\beta }}^{\left(m\right)}$ is the estimator of *β* in the *m*th replication of the experiment and *N* = 10000. The simulation results are summarized in Tables 2 and 3, where the condition number of *X*′*X*, that is, *κ* = *λ*_1_/*λ*_5_, is also given.

From the simulation results shown in Tables 2 and 3, we can see that, with the increase of the level of multicollinearity, the estimated MSE values of the three estimators increase in general. However, the proposed estimator SRPC behaves better than the competing estimators in most of the cases. In addition, the more severe the collinearity is, the more pronounced the superiority of SRPC is. Therefore, the proposed estimator is recommended when the explanatory variables are moderately or severely collinear.

## Figures and Tables

**Table 1:** Estimated MSE values of the OME, PCR, and SRPC.

	*β*_0_	*β*_1_	*β*_2_	*β*_3_	*β*_4_	MSE
${\hat{\beta }}_{\text{OME}}$	0.4014	0.5695	−0.1543	0.3402	0.1252	0.8202
${\hat{\beta }}_{\text{PCR}}$	−0.0182	0.5359	−0.0344	0.2838	0.2104	0.0461
${\hat{\beta }}_{\text{SRPC}}$	0.0885	0.5566	−0.0201	0.2641	0.1739	0.0398

**Table 2:** Estimated MSE with *n* = 100.

	*γ* = 0.9		*γ* = 0.99		*γ* = 0.999		*γ* = 0.9999	
	*W* = 2	*W* = 5	*W* = 2	*W* = 5	*W* = 2	*W* = 5	*W* = 2	*W* = 5
${\hat{\beta }}_{\text{OME}}$	0.1419	0.3463	2.6338	5.0907	11.884	12.911	129.02	220.67
${\hat{\beta }}_{\text{PCR}}$	0.0948	0.0936	0.8798	0.8825	8.7618	8.7304	87.503	85.314
${\hat{\beta }}_{\text{SRPC}}$	0.0994	0.1190	0.7177	1.1484	5.8144	6.7482	55.871	55.936
*κ*	21.4		123.8		1230.4		12326.7	

**Table 3:** Estimated MSE with *n* = 50.

	*γ* = 0.9		*γ* = 0.99		*γ* = 0.999		*γ* = 0.9999	
	*W* = 2	*W* = 5	*W* = 2	*W* = 5	*W* = 2	*W* = 5	*W* = 2	*W* = 5
${\hat{\beta }}_{\text{OME}}$	0.3155	0.3250	3.3823	4.0796	25.185	46.592	347.39	348.43
${\hat{\beta }}_{\text{PCR}}$	0.1629	0.1610	1.5253	1.4910	14.818	14.815	146.88	149.92
${\hat{\beta }}_{\text{SRPC}}$	0.1470	0.2053	0.8802	1.4796	7.0234	8.1218	69.063	69.919
*κ*	21.6		275.3		1946.0		19348.4	
